# An *In-Vitro* Study for Early Detection and to Distinguish Breast and Lung Malignancies Using the Pcb Technology Based Nanodosimeter

**DOI:** 10.1038/s41598-018-36805-2

**Published:** 2019-01-23

**Authors:** P. Venkatraman, C. S. Sureka

**Affiliations:** Department of Medical Physics, Bharathiar University, Coimbatore, Tamil Nadu India

## Abstract

Since the early detection of cancer increases the chance of successful treatment, the present study focused to confirm the suitability of an indigenously fabricated multilayer PCB technology based 3D positive ion detector to detect breast and lung malignancy at an early stage. The 3D positive ion detector is a type of gas filled radiation detector works under the principle of ion induced ionization using an exempted micro curie activity source. Earlier studies report that malignant cells can be detected by analyzing the Volatile Organic Compounds (VOCs) exhaled by those cells that serve as eminent biomarkers for malignant detection. Based on this, the present study analyzed the signals produced in the detector by VOCs exhaled from 140 biopsy tissue samples that include tissue of normal and all stages of breast and lung malignancy. To strengthen the present data, the normal and advanced breast and lung malignant tissues were also analyzed using the Gas Chromatography- Mass Spectrometry (GC-MS). From this study, it is confirmed that the present 3D positive ion detector can be used to detect both breast and lung malignancy and also to distinguish them based on the variation in four basic physical parameters of the output pulse such as frequency, amplitude, rise time and fall time and four derived parameters of the pulse such as FWHM, area of the pulse, ionization cluster size, and ion drift time.

## Introduction

According to the World Health Organization (WHO) cancer fact 2017, cancer is the second leading cause of death (Cardio-vascular disease is the first cause of death in most of the population) and it causes nearly 1 in 6 deaths globally. Approximately, 70% of cancer deaths are recorded in low and middle economic countries. This is generally due to the growth, and age of the population, lifestyle includes smoking, and drinking alcohol, poor diet, physical inactivity, lack of immunity, infections etc.,

The International Agency for Research on Cancer (IARC) estimated 14.1 million new cancer cases and 8.2 million cancer deaths worldwide in 2012. The WHO estimated 8.8 million cancer deaths in 2015 which included cancer of lung, liver, colorectal, stomach, and breast in the order of 1.690, 0.788, 0.774, 0.754, and 0.571 million respectively. However, the IARC expected the global cancer burden of up to 21.7 million new cancer cases and 13 million cancer deaths by 2030^1^. Breast cancer in females and lung cancer in males are the most frequently diagnosed cancers and the leading cause of cancer death for each sex in both economically developed and developing countries, except lung cancer is preceded by prostate cancer as the most frequent cancer among males in economically developed countries. Breast malignancy is the second leading cause of deaths in women^2^. It is estimated that the chances of 5 years survival rate of breast malignant patients were 96% in localized condition and 86% in metastases stage in 2017^3^, which increased to 98.3% in localized condition^4^. Hence, early detection of breast malignancy is the urgent need of today to decline the rate of mortality^5^. However, lung malignancy is the fifth leading cause of deaths both in men and women^6^. In 2012, a total of 1.8 million new lung cases were estimated that accounts for 12.9% of all new cancer diagnoses. According to the Global Burden of Disease study 2020^7^, the five-year survival rate (17.8%) of lung cancer was much lower than that of other leading cancers.

In general, an invasive technique such as biopsy and non-invasive techniques such as mammography, ultrasound, Magnetic Resonance Imaging (MRI), Computed Tomography (CT) scan, Positron Emission Tomography (PET) scan are the common methods used for the detection of breast and lung malignancy. In spite of effective and frontline screening tool, the sensitivity of mammography significantly decreases in the case of heterogeneously dense breast malignancy. It failed to diagnose almost half of the palpable tumours in extremely dense breasts cases.

However, many researchers manifests that Magnetic resonance and ultrasound imaging are quite good approaches to detect malignant and invasive malignancy as compared to mammography. These imaging strategies are supposed to overcome the drawbacks of mammography. However, few researchers have shown that these screening methods result in over diagnosis, leading to increased recall and negative open surgical biopsy rates^5,6^. The molecular screening methods in combination with these imaging techniques are helpful in alleviating this situation^8,9^.

In addition to these techniques, biomarker technique is an innovative approach emerging in the last decade, which can detect malignancy in early stage too^6^. This technique is also useful for monitoring the progression and regression of malignant after treatment. However, the biomarker technique of malignant detection involves the search of tissues and bio-fluids. The biomarkers may be genes, proteins, metabolites and breathing compounds which provide information’s about the abnormal growth of cells present in the malignant region.

To overcome the issues arised in detecting these biomarkers various techniques such as chromatography, immunohistochemistry (IHC), enzymelinked immunosorbent assay; (ELISA), two dimensional gel electrophoresis (2DE), fluorescence *in situ* hybridization (FISH), polymerase chain reaction (PCR), real-time polymerase chain reaction (RT-PCR), matrix-associated laser desorption/ionization time-of-flight MS (MALDI-TOF-MS), surface-enhanced laser desorption/ionization time-of-flight MS (SELDI-TOF-MS), liquid chromatography coupled with various detectors^10–13^ have been employed to detect the malignant cells.

Among these techniques, gas chromatography is considered to be the most appropriate and best approach due to its ease of operation, selectiveness, and reproducibility and low limits of detection. Studies reported that the GC- MS based metabolomics can be used to identify specific volatile organic compounds (VOCs) which serve as eminent source biomarkers for malignant diagnosis^14^. But, the identification or structural elucidation of these VOC molecules is limited due to the lack of universal metabolite- specific libraries. These drawbacks would be rectified by the emergence of various novel technologies viz., comprehensive two-dimensional gas chromatography (GC × GC) paired with time-of-flight mass spectrometry (TOF-MS), improvement in mass spectrometer instrumentation and in algorithms for identification, development of online opened metabolomics databases^15^ etc. The development of nano-liquid chromatography on micro-chips is an innovation in gas chromatography as it requires samples at nano gram level and provides detection at nano or low levels^16–18^. However, the GC- MS technique has various limitations such as immobile system, slow, need for pre-concentration, requirement of known compound quantification, impossible for real-time measurements, compounds should be thermally stable, not suitable for clinical use etc.

Earlier studies reported that lungs emit various VOCs include Benzene, Ethylbenzene, Cyclohexane, methanol, ethanol, dodecane and tridecane, and the breast emit alkanes, alkenes, ketones, halogenated hydrocarbons, aldehydes, alcohols, esters, unsaturated hydrocarbons, terpenes, siloxanes, and aromates^19–32^.

In the present work, we have put forth an attempt to confirm the suitability of an indigenously fabricated multilayer PCB (Printed Circuit Board) technology based hole type 3D positive ion detector to detect breast and lung malignancy by analysing its signal variation that occurs due to variation in the emission of VOC from malignant cells. Basically, this detector combines the working principle of thick gas electron multiplier (THGEM)^33^ and resistive plate counter as presented by Bashkirov *et al*.^34–38^. In continuation to their research, we improved the efficiency of the detector by updating the detector structure and the same was confirmed as a gas sensor in addition to other applications^39^. Based on the characteristics of the 3D positive ion detector as gas sensor, the present data were obtained by collecting the VOC emitted by both normal and malignant breast and lung tissues of all stages such as stage 0 (Cancer hasn’t spread), stage 1 (corresponds to local stage), stage 2 (corresponds to either local or regional stage depending on lymph node involvement), stage 3a (corresponds to regional stage), stage 3b (may have spread to lymph nodes near the breastbone) and stage 4 (Cancer has spread beyond the lymph nodes into other parts of the body) over a range of pressure (1 to 10 Torr). In order to confirm the differential emission of VOCs between normal and malignant tissue, the Gas Chromatography- Mass Spectrometry (GC-MS) spectrum of both normal and malignant (stage 4) breast and lung tissue has also been included.

## Materials and Methods

### Sample collection

10 mg of normal and malignant breast and lung tissue of all stages were collected from concerned adult and senior patients of both sexes who were evaluated in a biopsy centre and enrolled to use their tissue specimen for clinical research. Patients with active and/or uncontrolled fungal infections, with uncertain etiology of febrile episode, with Human Immunodeficiency Virus (HIV), active or chronic hepatitis (i.e., quantifiable HBV-DNA and/or positive HbsAg, quantifiable HCV-RNA) or known history of HCV or HBV without documented resolution, tested for hepatitis B or other infections were excluded from this study. In addition, all antibiotics taken by the patients should be completed at least a week before to collect their sample. Then, the collected biopsy samples were stored with formaldehyde solution for further analysis.

### Experimental setup

**Figure 1:**
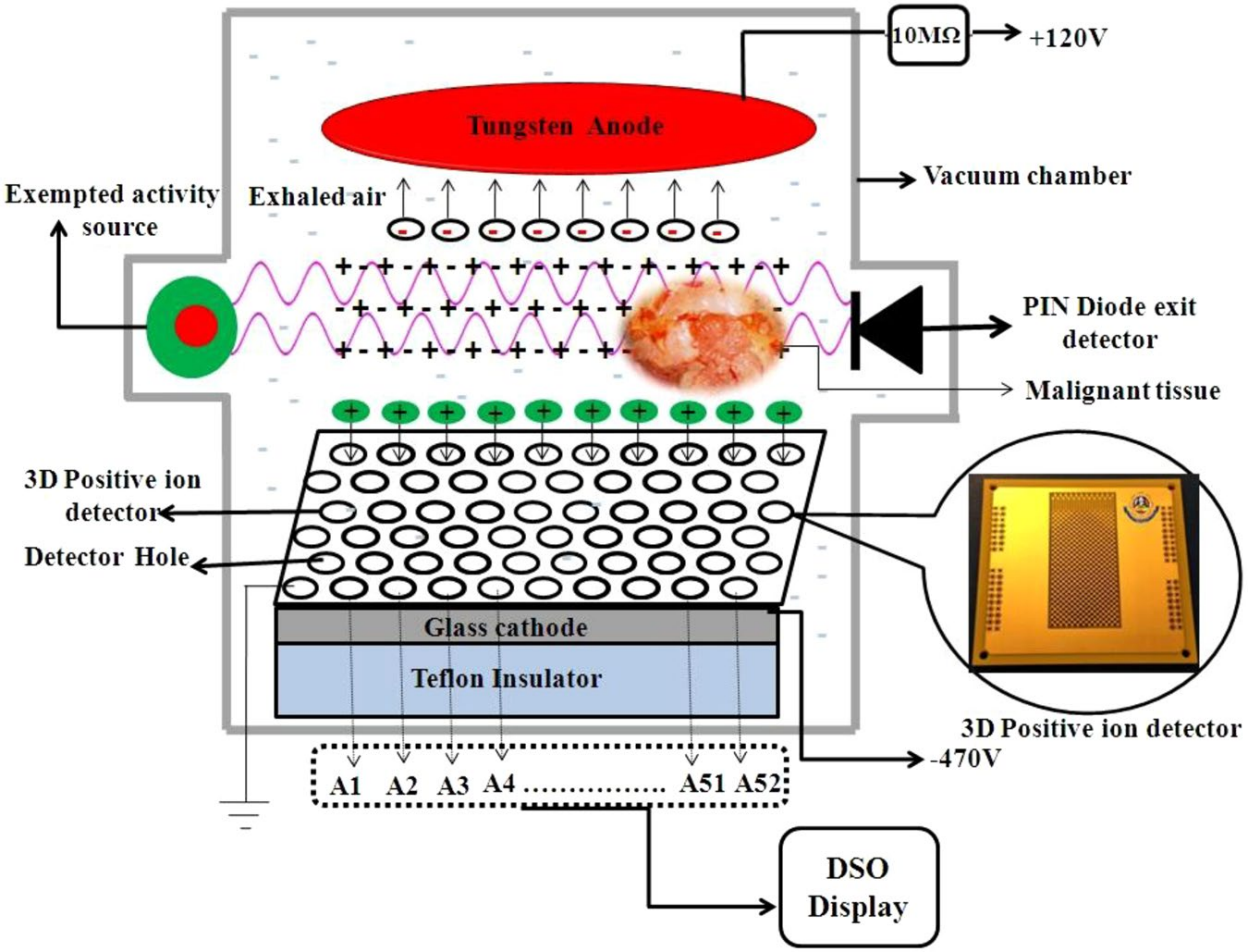
Schematic representation of the 3D positive ion detector

The indigenously developed Printed Circuit Board (PCB) technology based 3D positive ion detector was used to perform the present study. The basic structure, working principle, characteristics, and possible applications of the detector were reported in the previous article^39^. To short, the experimental setup consists of: (i) a vacuum chamber where the 3D positive ion detector with cathode and anode assembly, source and PIN diode were placed as shown in Fig. 1, (ii) vacuum system to evacuate the chamber, (iii) high voltage power supply, (iv) meters to measure temperature and pressure, and (v) a four channel Digital Storage Oscilloscope (DSO) to capture the signal. One of the DSO channel was connected to the anode which collects the anode signal induced by primary electrons. The PIN diode signal was captured in another channel through a timing filter amplifier and preamplifier setup to detect the primary particles traversing the gas medium. The remaining two channels were connected to the X and Y layer of the 3D positive ion detector through 1 MΩ resistance to record the signal produced by secondary electrons.

Initially, the chamber was evacuated to remove all the molecules present inside the chamber. Then, the normal lung and breast tissues were placed independently inside the chamber. It was allowed to exhale molecules from the tissue at an appropriate pressure ranging from 1 to 10 Torr. When the ionizing photons interact with the exhaled molecules, both electrons (primary) and positive ions of those molecules are produced. The primary electrons are captured by the anode and the positive ions drift into the detector holes. The positive ions produce avalanche of electrons (secondary) inside the detector hole by “ion induced ionization”. These secondary electrons are captured by the X and Y layer of the detector to produce signal in the DSO and it is recorded to analyse the amplitude, frequency, rise time, fall time and number of pulses of the signal. Then, the same experiment was performed for malignant tissues of stage 0, 1, 2, 3a, 3b and 4 independently and its DSO signal was recorded. All the measurements were repeated for 10 times for 140 tissue samples that include 10 sets of both normal and malignant breast and lung tissues at each stage.

### Sample preparation for GC-MS analysis

Sample preparation protocol reported previously^26–28^ has been utilized in this study with minor modifications. The sample has been processed in a 96 well plate which includes the following steps. As a primary step, the stock solution for myristic-d acid has been prepared with a concentration of 1 mg/mL which was used as an internal standard (IS). Then, 10 mg tissue of each case was mixed with 800 µL methanol followed by the addition of 20 µL internal standard (IS). Further this mixture has been vortexed and left on ice for 30 minutes. Then the mixture was centrifuged (Eppendorf Centrifuge 5804 C/R) for 20 minutes at 3,500 rpm in order to remove the precipitated protein. Clear supernatant was poured into the well plate and drawn through the solid phase under vacuum condition. Before extraction, the solid phase was activated with 600 µL methanol and 600 µL deionized water (Milli Q). After loading the sample, the solid phase was washed with 300 µL of water and eluted with 600 µL methanol with eluates collected in 96 well collection plates. Finally, the eluate was evaporated under nitrogen (N_2_) at room temperature. Samples were dried in Eppendorf™ Concentrator (5301) for approximately 4–5 hours (or till they were completely dry) and stored at 4° C till further analysis.

For Sample derivatization, 50 µL methoxylamine hydrochloride in 15 µg/µL pyridine has been added and followed by mixing at 600 rpm (Eppendorf™ Thermomixer) for nearly 2 hours at 35° C. Further, 50 µL MSTFA (with 1% trimethylchlorosilane) were added by mixing it for 60 minutes at 70° C with the formation of trimethylsilyl derivatives. Then the sample was centrifuged at 14,000 rpm for 10 minutes. For GC-MS analysis, the clear supernatant was shifted quickly to inserters in the labelled GC vials by leaving the precipitate at the bottom.

### Procedure for GC-MS analysis

The GC-MS analysis was carried out on 7890 A GC fitted with a GC sampler 120 (PAL LHX-AG12 – Agilent Technologies) auto sampler and coupled to Agilent 7000 Triple Quad system. A fused-silica capillary GC column, HP-5MS 30 m × 0.25 mm ID, chemically bonded with a 95% dimethylpolysiloxane 5% diphenyl cross-linked stationary phase (0.25 mm film thickness) was used as previously reported. The serum sample was inserted in the split less mode using helium as carrier gas. Initially the oven temperature was fixed at 40° C, and then increased to 300° C at a rate of 10° C per minute. After maintaining the temperature at 300° C for nine minutes, it was further increased to 305° C for one minute. Retention time was locked to the internal standard at 15.168 minutes. Electron impact ionization (EI) was used as an ionization source for the GC-MS analysis at 70 eV. GC-MS analysis was performed according to the GC parameters also described previously. Data acquisition was done in full scan mode from 0–650 *m*/*z* in 0.5 seconds scan time. Hexane was run between samples to remove contamination. Mass calibration was done with perfluorotributylamine (PFTBA). A quality control (QC) sample was run after every six samples of each batch of 23 samples during GC-MS analysis.

### Ethics Statement

In this study, the required biopsy tissues were collected from a nearby Biopsy Center with proper permission and informed consent from the participants. Most of the biopsy tissues (>98%) were used in a non-invasive manner to collect the VOCs exhaled by the tissues and returned to the same Biopsy Centre. A small portion of the remaining tissues were used to perform GC-MS analysis. All these procedures were carried out as per the Bharathiar University guidelines and regulations based on the approval of the Bharathiar University human ethical clearance committee with reference to the Ref No: BU/Med.Phys/Dr.CSS/Radiation Biology/Human Ethical Clearance/2016 dated 12.01.2016.

## Results and Discussion

### Confirm suitability of the PCB technology based positive ion detector to detect breast malignancy

**Figure 2:**
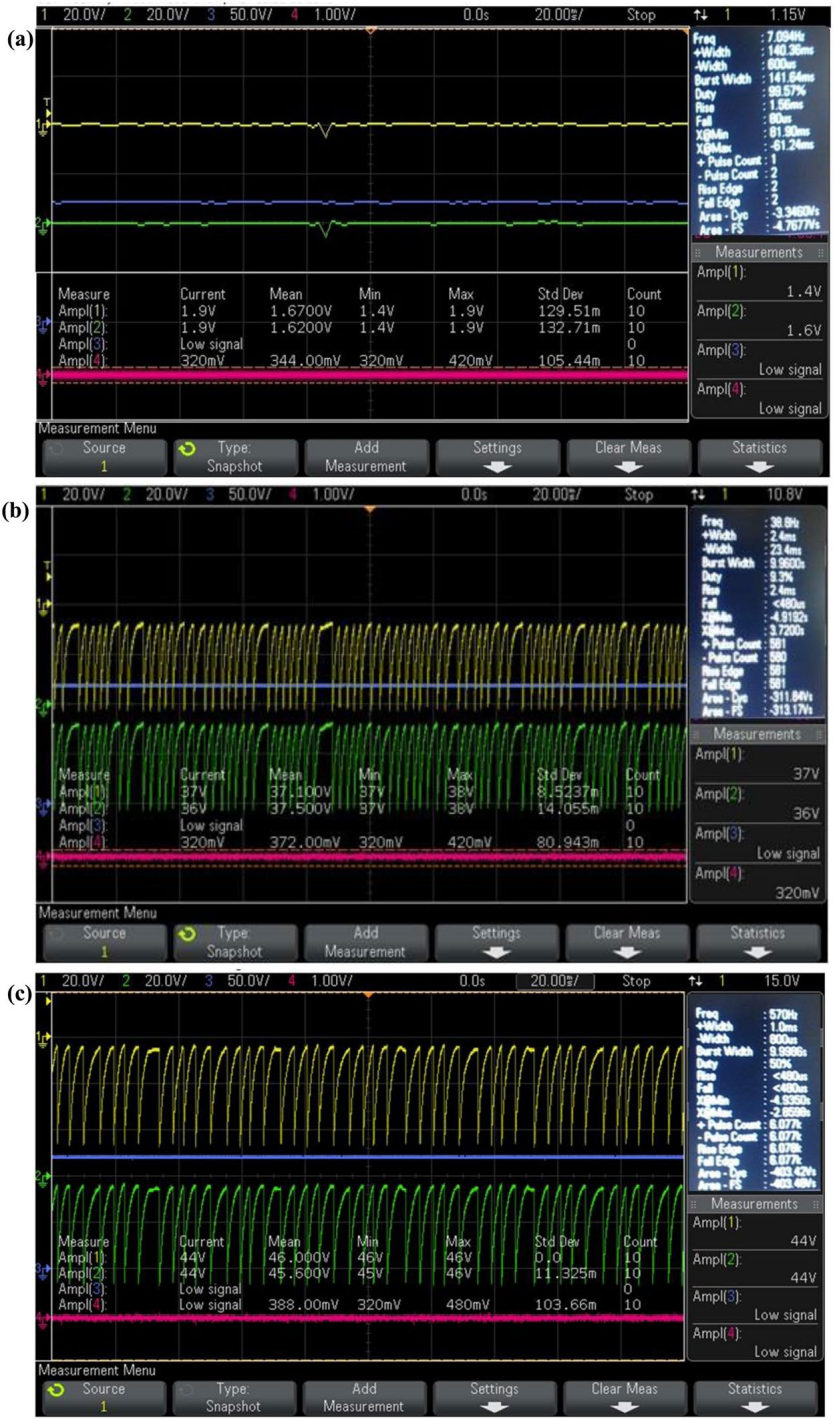
DSO signal captured by the 3D positive ion detector (**a**) without sample (**b**) in presence of normal breast tissues, and (**c**) Malignant breast tissue of stage 4.

As an initial step to confirm the signal captured by the 3D positive ion detector, + 120 V was supplied to the anode through 1 MΩ resistance and −470 V was supplied to the cathode of the detector and the DSO signal was captured in presence of the Co-60 source without placing the tissue inside the chamber. Then, the breast normal tissue followed by malignant breast tissue were placed inside the evacuated chamber to exhale the VOC molecules to the desired pressure and the DSO signals were captured. The DSO signals captured without sample, with normal and malignant breast tissue are shown in Fig. 2.

From the Fig. 2a, it is observed that the amplitude of the signal is 3.3 V (addition of both X and Y layer signal) in 20 ms without any sample. This was taken as noise and hence all the signal parameters obtained in presence tissue were subtracted from its corresponding noise value for further analysis. From the Fig. 2b,c, it is observed that the amplitude of the detector signal with normal and malignant breast tissue is increased many fold times. This implies that the detector can be used to analyze breast malignancy of all stages.

**Figure 3:**
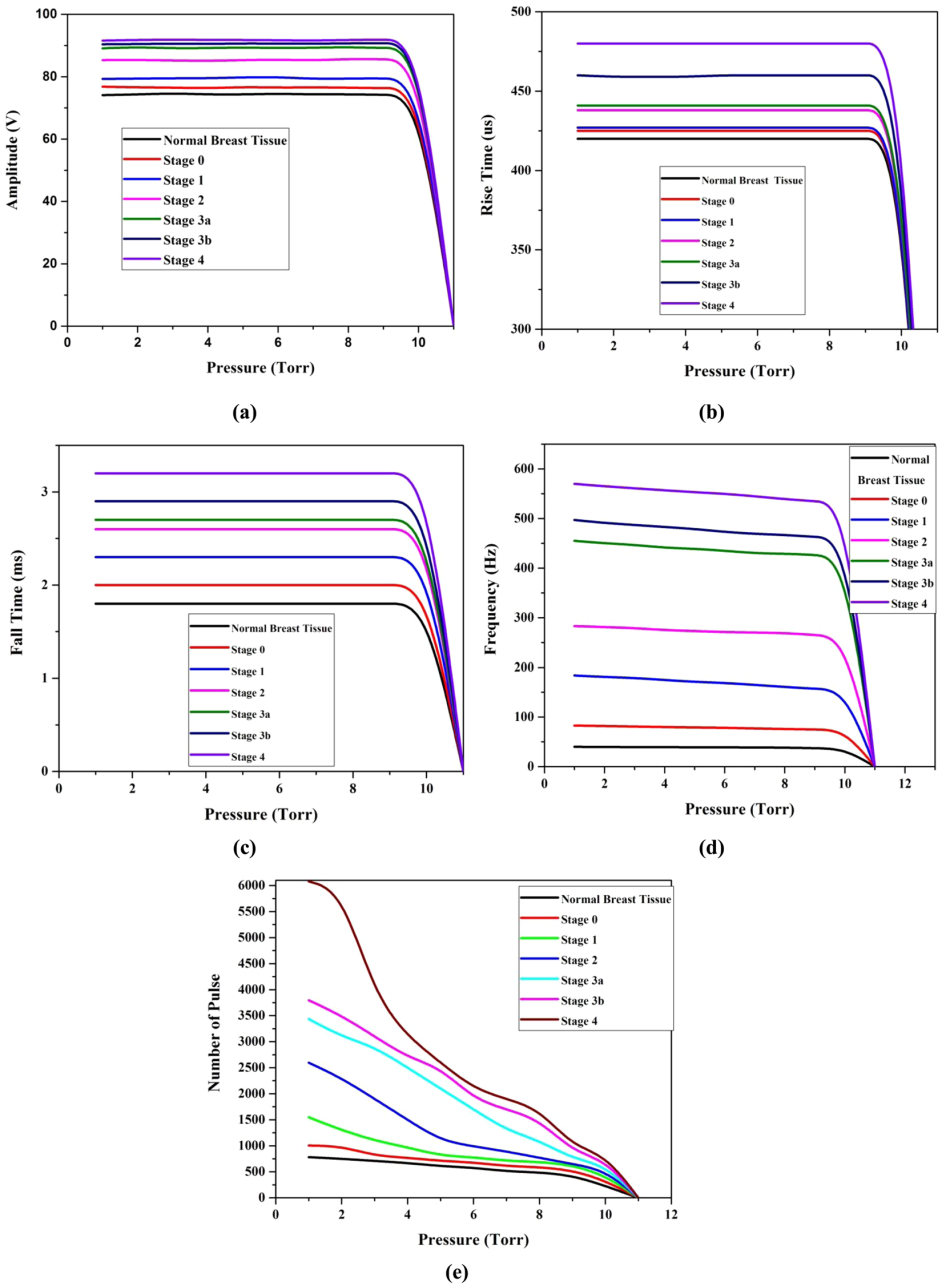
(**a**–**e**). Comparison of the signal amplitude, rise time, fall time, frequency, and number of pulse between normal and different breast malignant tissues medium.

Based on these, 10 sets of breast malignant tissue of stage 0, 1, 2, 3a, 3b and 4 were placed one by one and allowed to exhale the molecules to capture the signal. All the measurements were repeated for 10 times to improve the consistency in measurements. The mean amplitude, rise time, fall time, and frequency of pulses and number of pulses produced by normal and malignant breast tissue were compared and shown in Fig. 3(a–e). From the Fig. 3(a–e), it is observed that the signal amplitude, rise time, and fall time were constant over a range of pressure starts from 1 to 10 Torr and it increases gradually with respect to the stage of malignancy. It is also observed that there is a significant fall in frequency of pulses and number of pulses when the pressure of the gas molecules increases from 1 to 10 Torr. That is the mean frequency of the pulse emitted by normal, stage 0, 1, 2, 3a, 3b, 4 breast molecules was reduced as 38.4–36.0, 83.0–74.0, 184–155, 283–261, 455–420, 497–460, and 570–530 Hz respectively between 1–10 Torr. Similarly, the number of pulses was reduced as 782–230, 1007- 330, 1547- 430, 2598- 530, 3435- 630, 3795- 730, and 6079- 830 respectively between 1–10 Torr. All these signal parameters at 1 Torr pressure were compared among normal and breast malignant tissue of all stages and given in Table 1.

**Table 1:** Data taken from the 3D positive ion detector to confirm the breast malignancy.

Parameters/Breast		Without Sample	Normal	Stage 0	Stage 1	Stage 2	Stage 3a	Stage 3b	Stage 4
Amplitude (V)	Range	3.3–3.6	74.0–74.7	76.1–76.8	79.0–79.7	85.0–85.7	88.1–89.8	90.4–90.7	91.0–91.9
	Mean	3.57	74.4	76.5	79.3	85.4	89.3	90.5	91.5
	**б²**	0.0045	0.05	0.10	0.04	0.05	0.06	0.04	0.07
	**б**	0.067	0.22	0.32	0.22	0.23	0.25	0.21	0.27
	**%**	—	—	2.82	6.59	14.78	20.02	21.64	22.98
Frequency (Hz)	Range	7.0–7.1	36–38.8	83–84	182–184	279–282	453–455	493–495	568–570
	Mean	7.01	38.4	83.2	183.5	281.2	454.5	494.5	569.6
	**б²**	0.001	0.76	0.17	0.5	0.06	0.5	0.72	0.48
	**б**	0.03	0.87	0.42	0.707	0.03	0.707	0.84	0.7
	**%**	—	—	116.66	377.86	632.29	1083.59	1187.76	1383.33
Number of Pulse in 1 s	Range	1–3	781–782	1006–1008	1545–1547	2596–2598	3434–3435	3793–3795	6077–6079
	Mean	2.8	781.7	1007.5	1546.6	2597.5	3434.9	3794.6	6078.6
	**б²**	0.36	0.21	0.45	0.44	0.65	0.09	0.44	0.44
	**б**	0.63	0.483	0.7	0.67	0.85	0.32	0.69	0.69
	**Times**	—	—	2.88	9.78	23.22	33.94	38.54	67.76
Rise Time (µs)	Range	179–180	410–420	423–425	426–427	434–438	438–441	458–460	480–490
	Mean	179.8	418.9	424.5	426.7	437.2	440.5	459.6	485.6
	**б²**	0.177	0.98	0.5	0.23	0.16	0.94	0.26	0.178
	**б**	0.42	0.31	0.71	0.48	0.103	0.97	0.51	0.42
	**%**	—	—	1.33	1.86	4.37	5.15	9.71	15.92
Fall Time (ms)	Range	0.5–1.0	1.7–1.8	1.9–2.0	2.1–2.3	2.4–2.6	2.6–2.7	2.8–2.9	3.0–3.2
	Mean	0.86	1.77	1.96	2.22	2.55	2.69	2.89	3.15
	**б²**	0.03	0.002	0.002	0.005	0.0045	0.0009	0.0009	0.0045
	**б**	0.19	0.048	0.052	0.078	0.071	0.03	0.32	0.071
	**%**	—	—	10.73	25.42	44.07	51.97	63.27	77.96

NOP – Number of Pulses; б² - Variance; б - Standard Deviation.

From the Table 1, it is observed that the frequency, rise time, fall time and number of pulses of the signal have been increased gradually when the normal breast tissue converted into various stages of malignancy. For the stage 0, 1, 2, 3a, 3b and 4, the mean amplitude of the pulse produced by normal breast tissue was increased upto 2.82, 6.59, 14.78, 20.02, 21.64, and 22.98% respectively and the mean frequency of the signal emitted by normal breast tissue is shifted from 38.4 Hz to 83.2, 183.5, 281.2, 454.5, 494.5, and 569.6 Hz respectively. Then, the number of pulses detected for stage 0, 1, 2, 3a, 3b and 4 of breast malignancy is 2.88, 9.78, 23.22, 33.94, 38.54, and 67.76 times respectively higher than the pulses detected for normal breast tissue. Similarly, rise time and fall time of the stage 0, 1, 2, 3a, 3b and 4 of breast malignancy signal is 1.33, 10.73; 1.86, 25.42; 4.37, 44.07; 5.15, 51.97; 9.71, 63.27; and 15.92%, 77.96% respectively higher than that of normal breast tissue.

**Figure 4:**
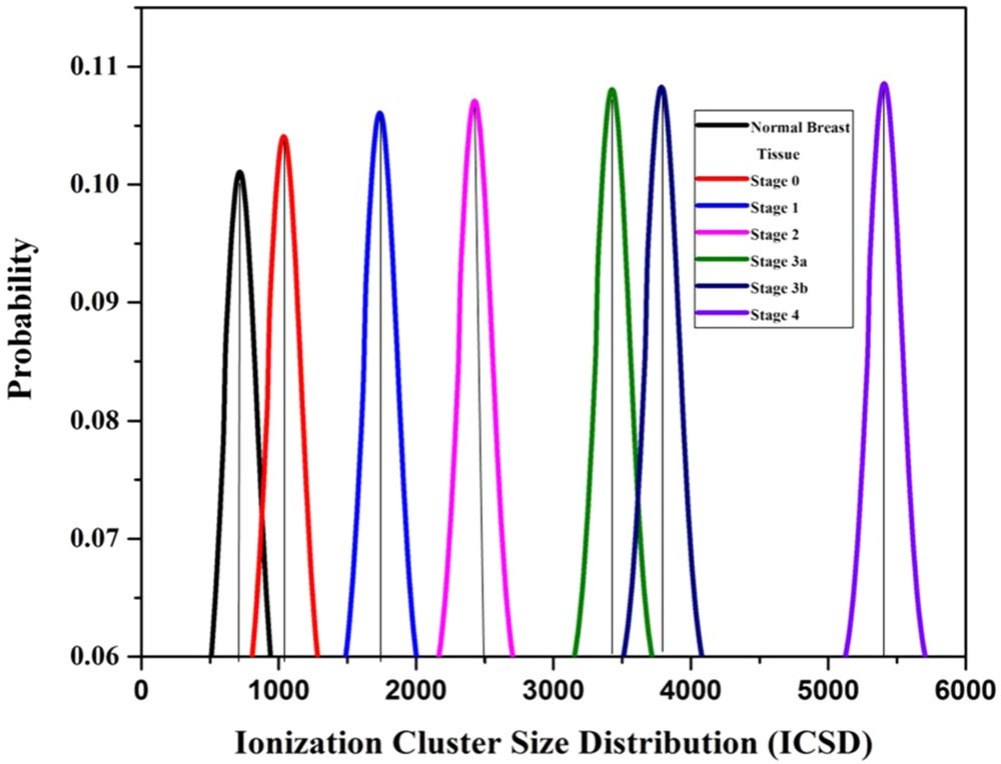
Ionization cluster size distribution under normal various breast malignant tissue media in presence of Co-60 source.

**Figure 5:**
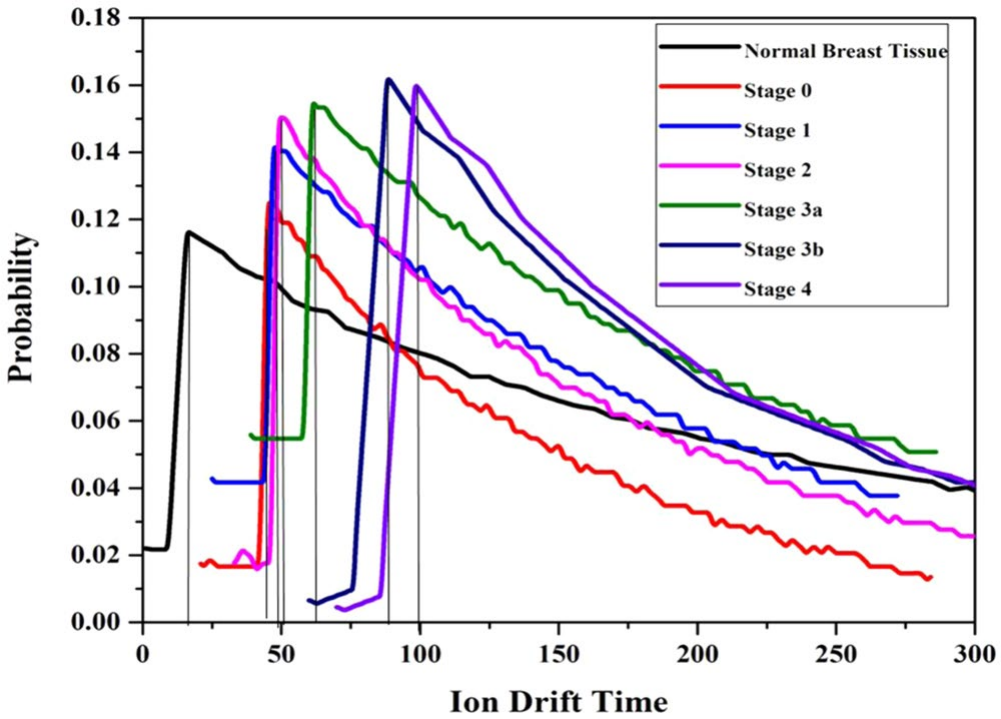
Ion Drift Time under normal various breast malignant tissue media in presence of Co-60 source.

In addition, there is a significant variation in the ICSD (Ionization Cluster Size Distribution) and ion drift time of the detector among normal and breast malignant tissues. It is shown in Figs 4, 5 respectively and data are tabulated in Table 2. From the Table 2, it is noted that the number of ionization produced by the VOC molecules exhaled by stage 0, 1, 2, 3a, 3b and 4 of breast malignancy is 4.12, 11.58, 26.41, 37.50, 41.87, and 75.37 times respectively higher than that of normal tissues. Similarly, the ion drift time in presence of stage 0, 1, 2, 3a, 3b and 4 of breast malignant tissue is also 15.92, 17.74, 20.29, 27.31, and 45.19, 50.04 times respectively higher than that of normal tissues.

**Table 2:** Ionization Cluster size distribution and Ion drift time analysis to confirm the breast malignancy.

Parameters/Breast		Without Sample	Normal	Stage 0	Stage 1	Stage 2	Stage 3a	Stage 3b	Stage 4
Ionization Cluster Size Distribution	Range	0–3	713–715	1008–1010	1542–1543	2601–2603	3339–3402	3768–3701	6100–6102
	Mean	2.6	714.7	1009.6	1542.8	2602.7	3395.5	3707.7	6101.8
	**б²**	0.84	0.41	0.44	0.16	0.41	0.85	0.81	0.36
	**б**	0.97	0.67	0.67	0.42	0.67	0.97	0.95	0.63
	**Times**	—	—	4.12	11.58	26.41	37.50	41.87	75.37
Ion Drift Time (µs)	Range	6–7	16.3–16.5	41–43	44–46	49–50	61–61.5	90–91	98–99
	Mean	6.9	16.47	42.7	45.7	49.9	61.45	90.9	98.9
	**б²**	0.09	0.0041	0.41	0.41	0.09	0.022	0.09	0.09
	**б**	0.32	0.067	0.67	0.67	0.32	0.16	0.32	0.32
	**Times**	—	—	15.92	17.74	20.29	27.31	45.19	50.04

ICSD- Ionization Cluster Size Distribution; IDT- Ion Drift Time; б² - Variance; б - Standard Deviation; NMR – Normal to Malignancy Ratio.

**Figure 6:**
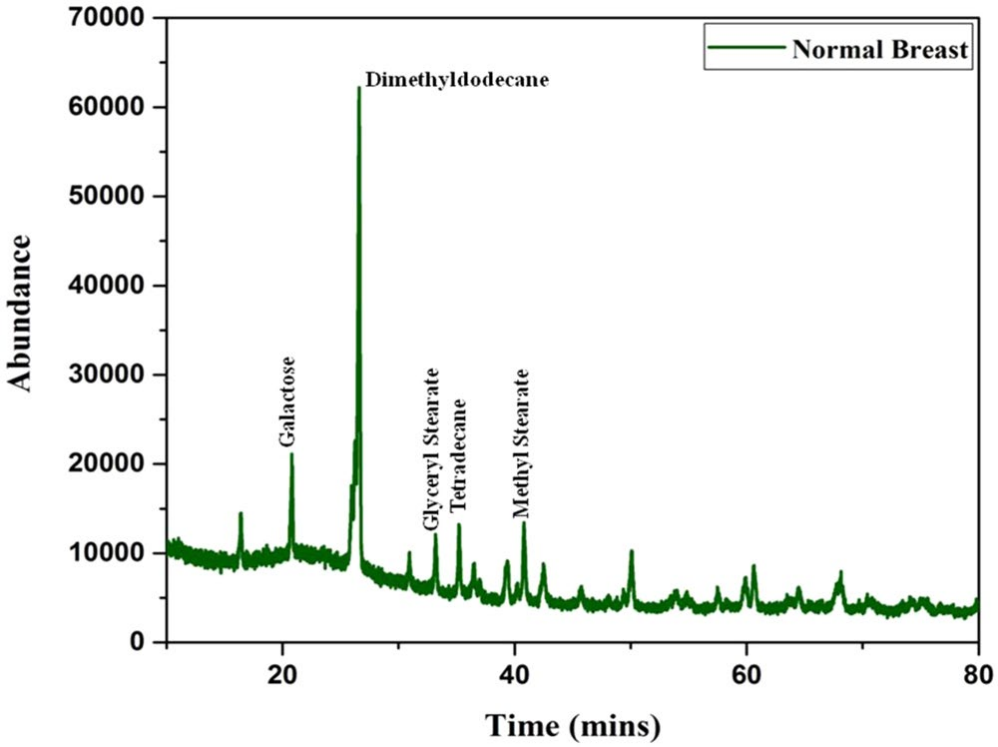
The typical total ion chromatograms (TICs) of breast healthy control.

**Figure 7:**
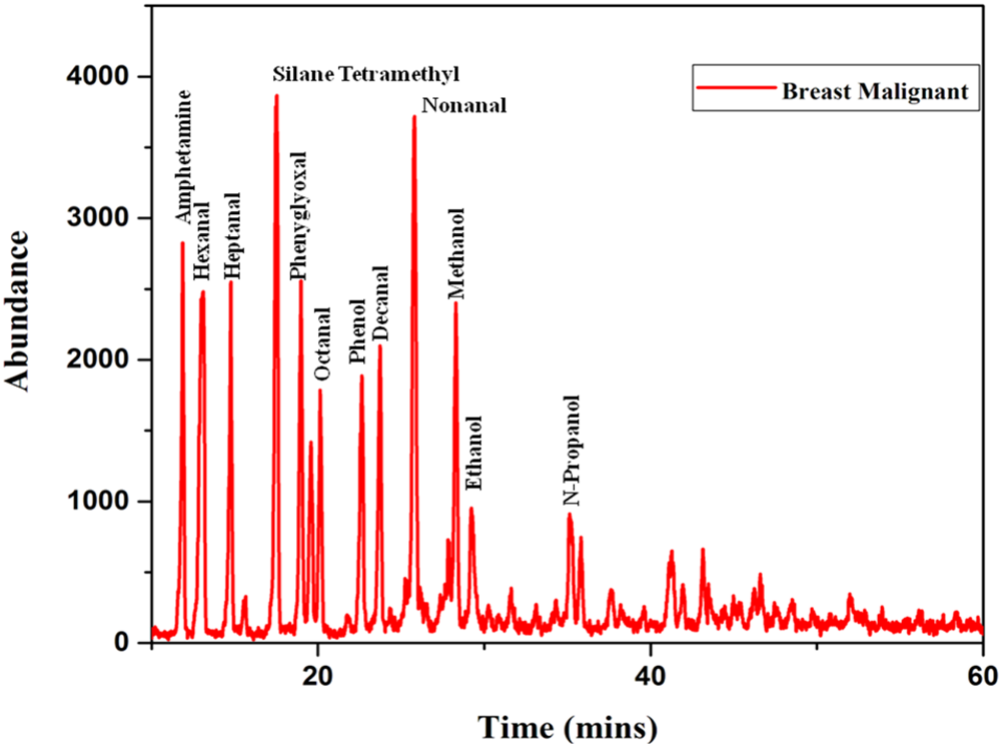
The typical total ion chromatograms (TICs) of breast malignant tissues.

From the data obtained from the indigenously fabricated 3D positive ion detector, it is observed that the number, composition and type of VOC molecules exhaled by normal and malignant breast tissues may be different. To confirm this, the GCMS of normal and stage 4 malignant breast tissues were taken and shown in Figs 6,7. From these Figs 6,7, it is observed that the normal breast tissue exhaled five VOCs namely Galactose, Dimethyldodecane, Glyceryl Stearate, Tetradecane, and Methyl Stearate. However, the stage 4 breast malignant tissue exhaled twelve VOCs namely Amphetamine, Hexanal, Heptanal, Silane Tetramethyl, Phenyglyoxal, Octanal, Phenol, Decanal, Nonanal, Methanol, Ethanol, and N-Propanol. It confirmed the observation of the 3D positive ion detector. Based on these, it is confirmed that the 3D positive ion detector can be used to diagnose and distinguish normal and breast malignancy of all stages.

### Confirm suitability of the PCB technology based 3D positive ion detector to detect lung malignancy

**Figure 8:**
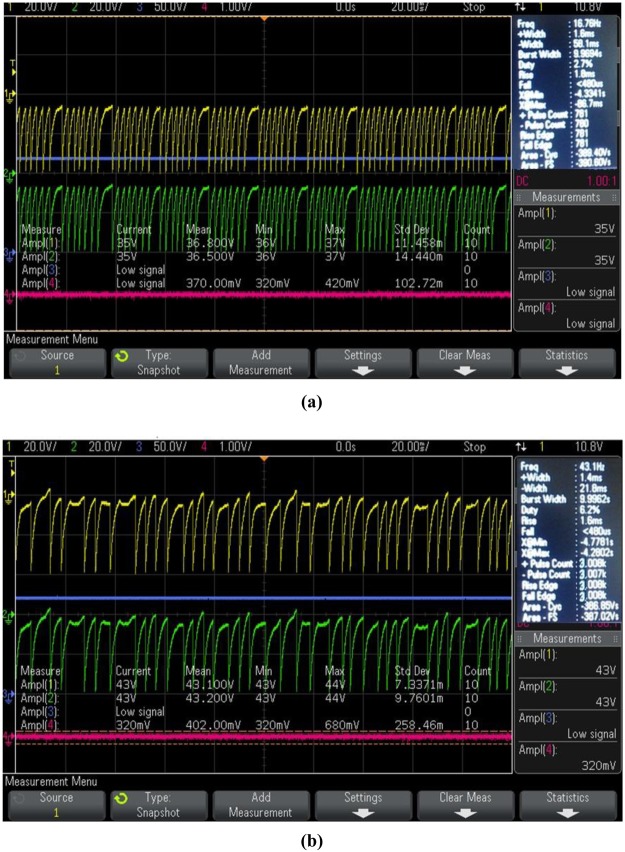
DSO signal captured by the 3D positive ion detector (**a**) in presence of normal lung tissues, and (**b**) Malignant lung tissue of stage 4.

As an initial step to confirm the signal captured by the 3D positive ion detector, +120 V was supplied to the anode through 1 MΩ resistance and −470 V was supplied to the cathode of the detector and the DSO signal was captured in presence of the Co-60 source without placing the tissue inside the chamber. Then, the lung normal tissue followed by malignant lung tissue were placed inside the evacuated chamber to exhale the VOC molecules to the desired pressure and the DSO signals were captured. The DSO signals captured with normal and malignant lung tissue are shown in Fig. 8.

From the Fig. 8a,b, it is observed that the amplitude of the detector signal with normal and malignant lung tissue is increased many fold times. This implies that the detector can be used to analyze lung malignancy of all stages.

**Figure 9:**
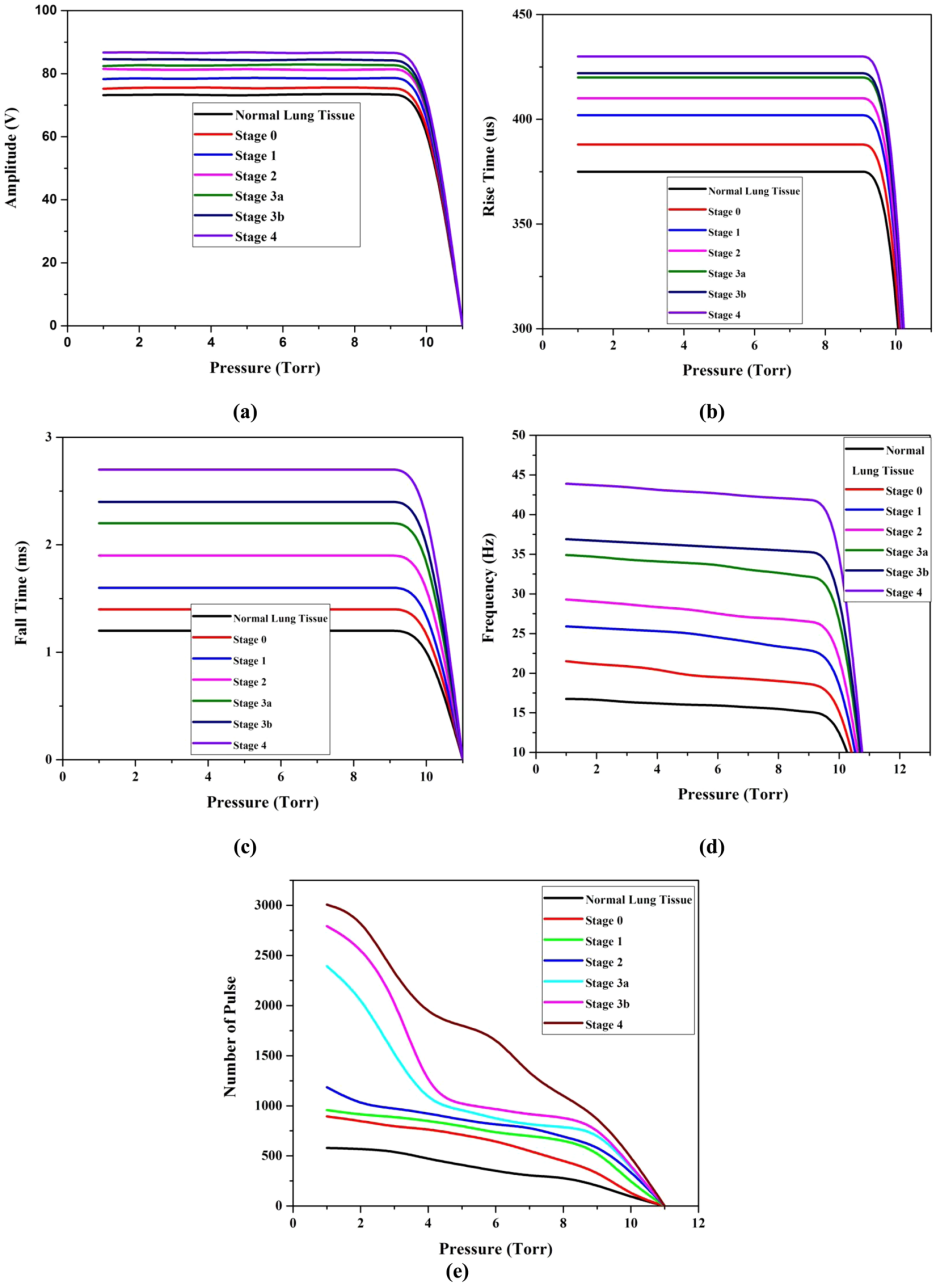
(**a**–**e**). Comparison of the signal amplitude, rise time, fall time, frequency, and number of pulse between normal and different lung malignant tissues medium in presence of Co-60.

Based on these, 10 sets of lung malignant tissue of stage 0, 1, 2, 3a, 3b and 4 were placed one by one and allowed to exhale the molecules to capture the signal. All the measurements were repeated for 10 times to improve the consistency in measurements. The mean amplitude, rise time, fall time, and frequency of pulses and number of pulses produced by normal and malignant lung tissue were compared and shown in Fig. 9(a–e). From the Fig. 9(a–e), it is observed that the signal amplitude, rise time, and fall time were constant over a range of pressure starts from 1 to 10 Torr and it increases gradually with respect to the stage of malignancy. It is also observed that there is a significant fall in frequency of pulses and number of pulses when the pressure of the gas molecules increases from 1 to 10 Torr. That is the mean frequency of the pulse emitted by normal, stage 0, 1, 2, 3a, 3b, 4 lung molecules was reduced as 16.6–14.9, 21.5- 18.0, 25.9- 22.0, 29.3- 26.1, 34.9- 31.9, 36.9- 35.0, and 43.9- 41.5 Hz respectively between 1–10 Torr. Similarly, the number of pulses was reduced as 580–90, 894- 110, 957- 230, 1185- 350, 2393- 390, 2793- 400, and 3008- 500 respectively between 1–10 Torr. All these signal parameters at 1 Torr pressure were compared among normal and breast malignant tissue of all stages and given in Table 3.

**Table 3:** Data taken from the 3D positive ion detector to confirm the lung malignancy.

Parameters/Lung		Without Sample	Normal	Stage 0	Stage 1	Stage 2	Stage 3a	Stage 3b	Stage 4
Amplitude (V)	Range	3.3–3.6	73.1–73.8	75.1–75.6	78.1–78.8	81.2–81.7	82.1–82.8	84.1–84.7	86.3–86.9
	Mean	3.57	73.4	75.2	78.4	81.4	82.3	84.5	86.5
	**б²**	0.0045	0.051	0.027	0.047	0.025	0.05	0.041	0.065
	**б**	0.067	0.22	0.16	0.21	0.15	0.22	0.20	0.25
	**%**	—	—	2.45	6.18	10.89	12.12	15.12	17.84
Frequency (Hz)	Range	7.0–7.1	16–16.76	20–21.3	24–25	27–29	33–34	35–36	43.0–43.1
	Mean	7.01	16.6	21.15	24.8	28.7	33.8	35.6	43.02
	**б²**	0.001	0.1	0.16	0.17	0.45	0.17	0.20	0.001
	**б**	0.03	0.3	0.41	0.42	0.67	0.42	0.51	0.04
	**%**	—	—	27.41	49.39	72.89	103.61	114.45	159.16
Number of Pulse in 1 s	Range	1–3	579–581	893–895	956–957	1185–1186	2393–2394	2794–2793	3008–3009
	Mean	2.8	580.5	894.6	956.7	1185.7	2393.8	2793.6	3008.7
	**б²**	0.36	0.45	0.48	0.21	0.21	0.16	0.24	0.21
	**б**	0.63	0.71	0.67	0.48	0.48	0.42	0.51	0.48
	**Times**	—	—	5.41	6.48	10.42	31.23	38.12	41.82
Rise Time (µs)	Range	179–180	373–375	382–388	400–402	407–410	417–420	421–422	429–430
	Mean	179.8	374.5	386.3	401.5	409.2	419.1	421.7	429.6
	**б²**	0.177	0.5	0.98	0.5	0.106	0.14	0.23	0.26
	**б**	0.42	0.71	0.99	0.7	0.103	0.19	0.48	0.51
	**%**	—	—	3.15	7.20	9.26	11.90	12.60	14.71
Fall Time (ms)	Range	0.5–1.0	0.9–1.2	1.3–1.4	1.5–1.6	1.8–1.9	2.1–2.2	2.3–2.4	2.5–2.7
	Mean	0.86	1.16	1.39	1.58	1.89	2.19	2.38	2.67
	**б²**	0.03	0.008	0.0009	0.0016	0.0009	0.0009	0.0016	0.0041
	**б**	0.19	0.096	0.032	0.42	0.032	0.0.032	0.042	0.067
	**%**	—	—	19.82	36.20	62.93	88.79	105.17	130.17

NOP – Number of Pulses; б² - Variance; б - Standard Deviation.

From the Table 3, it is observed that the frequency, rise time, fall time and number of pulses of the signal have also been increased gradually when the normal lung tissue converted into various stages of malignancy. For the stage 0, 1, 2, 3a, 3b and 4, the mean amplitude of the pulse produced by normal breast tissue was increased upto 2.45, 6.18, 10.89, 12.12, 15.12, and 17.84% respectively and the mean frequency of the signal emitted by normal lung tissue is shifted from 16.6 Hz to 21.15, 24.8, 28.7, 33.8, 35.6, and 43.02 Hz respectively. Then, the number of pulses detected for stage 0, 1, 2, 3a, 3b and 4 of lung malignancy is 5.41, 6.18, 10.42, 31.23, 38.12, and 41.82 times respectively higher than the pulses detected for normal lung tissue. Similarly, rise time and fall time of the stage 0, 1, 2, 3a, 3b and 4 of lung malignancy signal is 3.15, 19.82; 7.20, 36.20; 9.26, 62.93; 11.90, 88.79; 12.60, 105.17; and 14.71%, 130.17% respectively higher than that of normal lung tissue.

**Figure 10:**
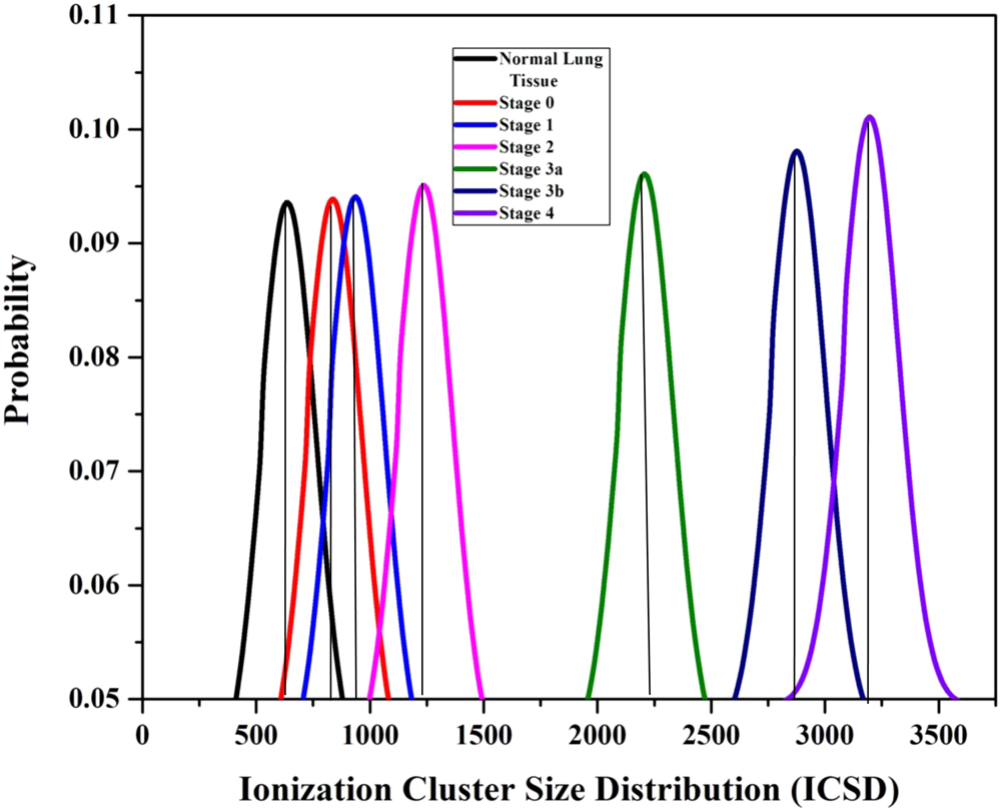
Ionization cluster size distribution under normal various lung malignant tissue media in presence of Co-60 source.

**Figure 11:**
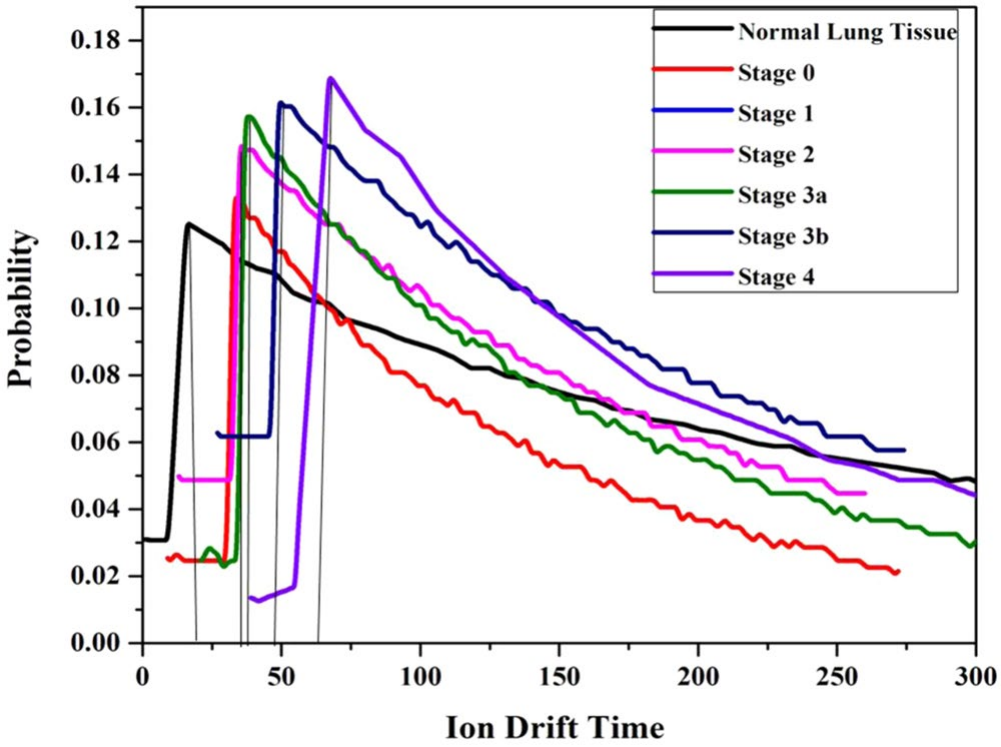
Ion Drift time under normal various lung malignant tissue media in presence of Co-60 source.

In addition, there is a significant variation in the ICSD (Ionization Cluster Size Distribution) and ion drift time of the detector among normal and lung malignant tissues. It is shown in Figs 10,11 respectively and data are tabulated in Table 4. From the Table 4, it is noted that the number of ionization produced by the VOC molecules exhaled by stage 0, 1, 2, 3a, 3b and 4 of lung malignancy is 6.81, 8.72, 10.80, 33.41, 40.98, and 48.54 times respectively higher than that of normal tissues. Similarly, the ion drift time in presence of stage 0, 1, 2, 3a, 3b and 4 of lung malignant tissue is also 10.1, 12.5, 13.7, 21.3, 31.9, and 33.9 times respectively higher than that of normal tissues.

**Table 4:** Ionization Cluster size distribution and Ion drift time analysis to confirm the lung malignancy.

Parameters/Lung		Without Sample	Normal	Stage 0	Stage 1	Stage 2	Stage 3a	Stage 3b	Stage 4
Ionization Cluster Size Distribution	Range	0–3	528–530	890–891	990–992	1100–1102	2299–2300	2700–2701	3100–3101
	Mean	2.6	529.7	890.9	991.7	1101.8	2299.8	2700.9	3100.9
	**б²**	0.84	0.41	0.09	0.41	0.36	0.16	0.09	0.09
	**б**	0.97	0.67	0.32	0.67	0.63	0.42	0.32	0.32
	**Times**	—	—	6.81	8.72	10.80	33.41	40.98	48.54
Ion Drift Time (µs)	Range	6–7	15–16	31.5–32	35–36	37–38	49–50	65–67	69–70
	Mean	6.9	15.9	31.9	35.9	37.8	49.9	66.7	69.9
	**б²**	0.09	0.09	0.04	0.09	0.16	0.09	0.41	0.09
	**б**	0.32	0.32	0.21	0.32	0.42	0.32	0.67	0.32
	**Times**	—	—	10.1	12.5	13.7	21.3	31.9	33.9

ICSD- Ionization Cluster Size Distribution; IDT- Ion Drift Time; б² - Variance; б - Standard Deviation; NMR – Normal to Malignancy Ratio.

**Figure 12:**
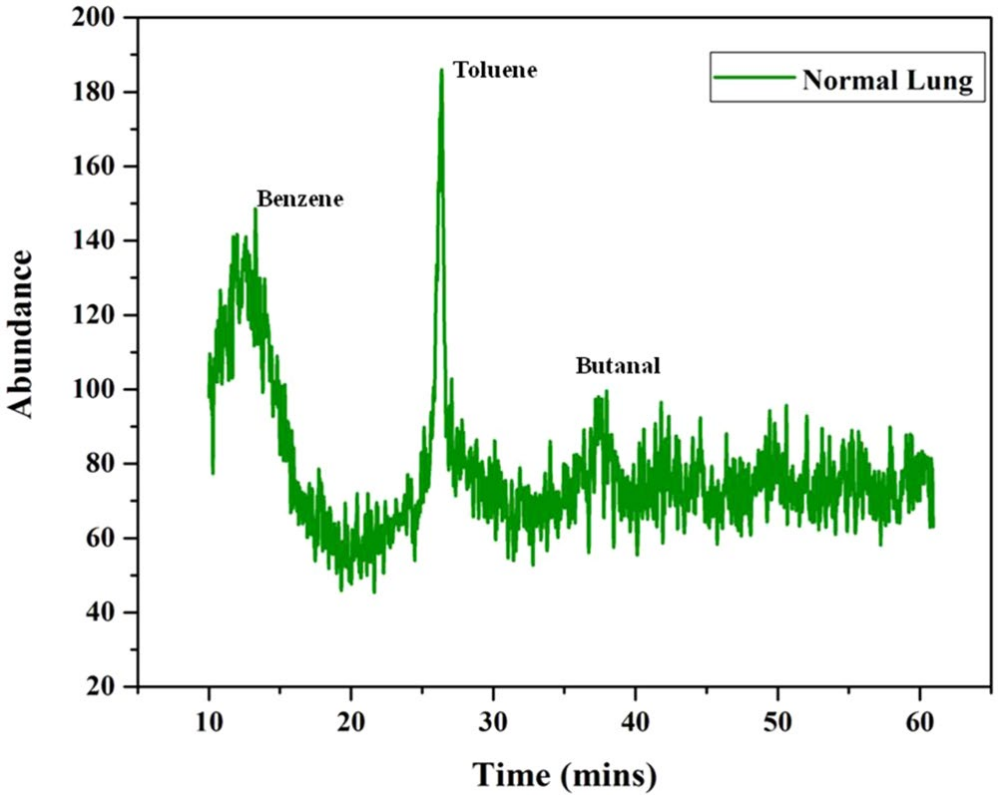
The typical total ion chromatograms (TICs) of lung healthy control.

**Figure 13:**
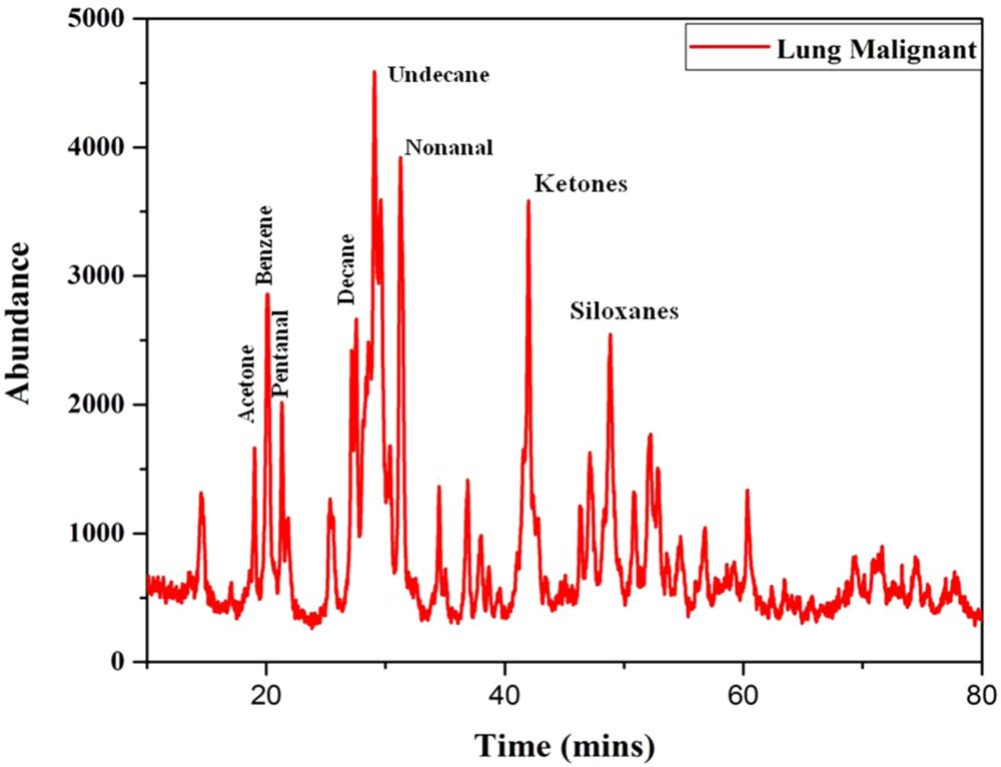
The typical total ion chromatograms (TICs) of lung malignant tissues.

From the data obtained from the indigenously fabricated 3D positive ion detector, it is observed that the number, composition and type of VOC molecules exhaled by normal and malignant lung tissues may be different. To confirm this, the GCMS of normal and stage 4 malignant lung tissues were taken and shown in Figs 12,13. From these Figs 12,13, it is observed that the normal lung tissue exhaled three VOCs namely Benzene, Toluene, and Butanal. However, the stage 4 lung malignant tissue exhaled eight VOCs namely Acetone, Benzene, Pentanal, Decane, Undecane, Nonanal, Ketones, and Siloxanes. It confirmed the observation of the 3D positive ion detector. Based on these, it is confirmed that the 3D positive ion detector can be used to diagnose and distinguish normal and lung malignancy of all stages.

### Comparison of signal between breast and lung malignancy

**Figure 14:**
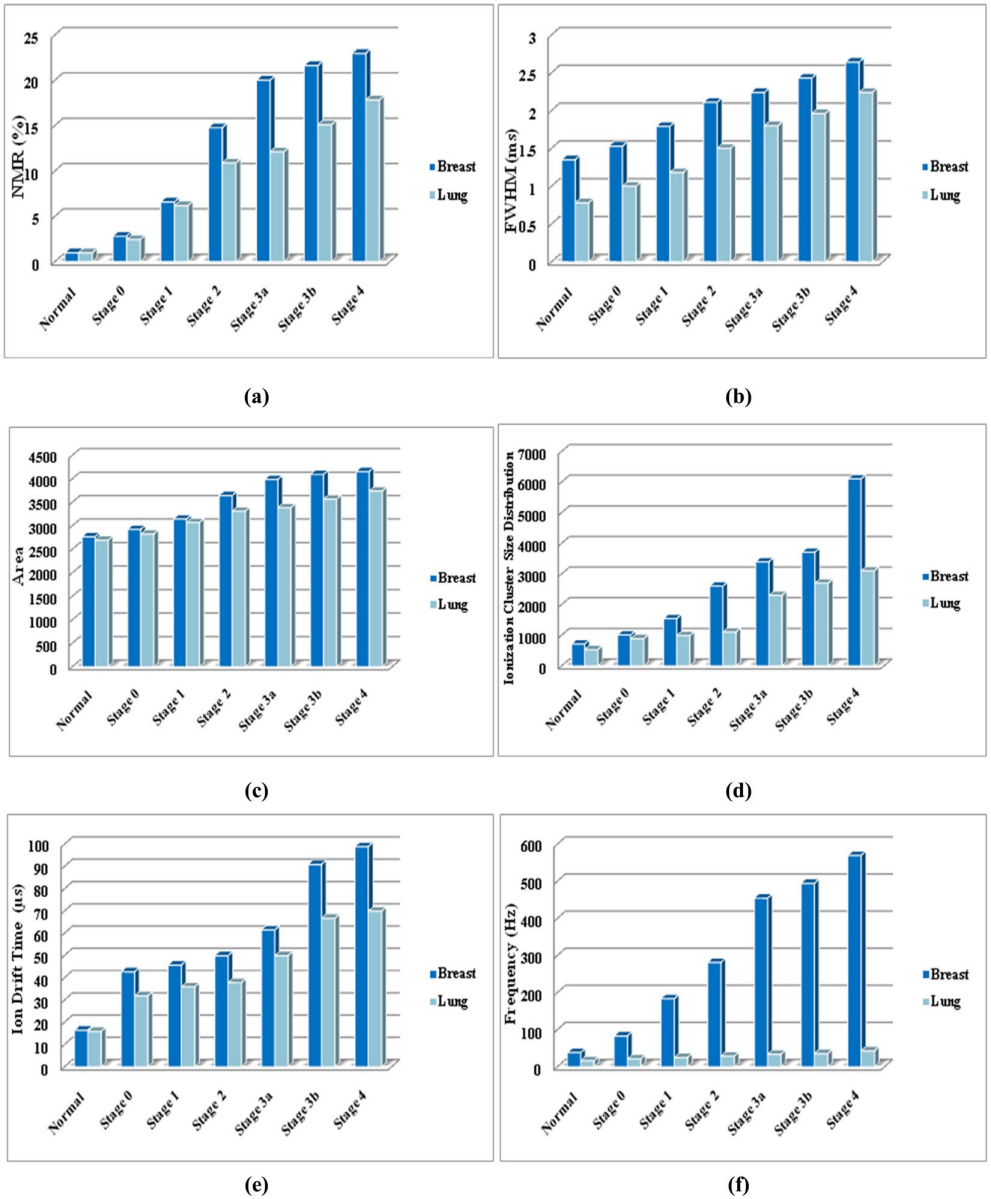
Comparison of signal between breast and lung malignancy through its (**a**) NMR, (**b**) FWHM, (**c**) Area of the pulse, (**d**) Ionization cluster size, (**e**) Ion drift time, and (**f**) Frequency.

Followed by the confirmation of the 3D positive ion detector to diagnose breast and lung malignancies of all stages, the potential application of the detector to distinguish those malignancies was also analyzed using the same data sets. To do so, the Normal to Malignant Ratio (NMR) was calculated from the amplitude of the normal and malignant tissues. Similarly, Full Width Half Maximum (FWHM) and area of the individual pulses were calculated from the amplitude, rise time and fall time of the signal. The remaining important parameters such as frequency, ICSD and IDT were taken for comparison and tabulated in Table 5 and also presented as bar chart in Fig. 14 for better understanding.

**Table 5:** Comparison of signal parameters between breast and lung malignancy.

Parameter		Normal	Stage 0	Stage1	Stage2	Stage3a	Stage3b	Stage4	
NMR (%)		B	1	2.82	6.59	14.78	20.02	21.64	22.98
		L	1	2.45	6.18	10.89	12.12	15.12	17.84
		%	0	15.10	6.63	35.72	65.18	43.12	28.81
Size of the pulse	FWHM (ms)	B	1.35	1.53	1.79	2.11	2.24	2.43	2.64
		L	0.78	1.00	1.18	1.5	1.8	1.96	2.24
		%	73.07	53.00	51.69	40.6	24.4	23.97	17.85
	Area (V^2^)	B	2767.6	2926.1	3144.2	3646.3	3987.2	4095.1	4156.1
		L	2693.7	2827.5	3073.2	3312.9	3386.6	3570.1	3741.1
		%	2.74	3.48	2.31	9.84	17.73	14.70	11.09
Ionization Cluster Size Distribution		B	714.7	1009.6	1542.8	2602.7	3395.5	3707.7	6101.8
		L	529.7	890.9	991.7	1101.8	2299.8	2700.9	3100.9
		%	34.92	13.32	55.57	136.22	47.647	37.27	96.77
Ion Drift Time (µs)		B	16.47	42.7	45.7	49.9	61.45	90.9	98.9
		L	15.9	31.9	35.9	37.8	49.9	66.7	69.9
		Times	0.35	3.38	2.72	3.20	2.31	3.62	4.14
Frequency (Hz)		B	38.4	83.2	183.5	281.2	454.5	494.5	569.6
		L	16.6	21.15	24.8	28.7	33.8	35.6	43.02
		%	131.32	293.38	639.91	879.79	1244.67	1289.0	1224.0

From the Table 5 and Fig. 14, it is observed that the breast malignancy shows all the signal parameters higher than lung malignancy at all stages. This may be due to the high rate of emission of VOCs from breast than lung because of its high rate of proliferation. The NMR between the breast and lung malignancy is varied considerably at all stages. The average range of frequency of the pulse for breast and lung is 38.4–569.6 and 16.6–43.02 Hz respectively that showed an independent spectrum of pulses exhaled by breast and lung malignant tissue. Hence, the frequency of pulse may be considered as one of the important parameters to distinguish breast and lung malignancy. Similarly, the average range of FWHM for breast and lung is 1.35 ms–2.64 ms and 0.78 ms–2.24 ms respectively and the average range of area under the individual pulse for breast and lung malignancy is 2767.6 V^2^–4156.1 V^2^ and 2693.7 V^2^–3741.1 V^2^ respectively, the average range of ICSD for breast and lung malignancy is 714.7–6101.8 and 529.7–3100.9 respectively, and the average range of IDT for breast and lung is 16.47–98.9 and 15.9–69.9 ms respectively. From this observation, it is confirmed that the 3D positive ion detector can be used for early detection of breast and lung malignancy and also to distinguish them.

## Conclusion

We have analysed the suitability of the indigenously fabricated multilayer PCB technology based 3D positive ion detector to detect breast and lung malignancy at an early stage by collecting the VOCs exhaled by normal and malignant breast and lung biopsy tissue of all stages. To confirm the differential emission of VOCs between normal and malignant tissue, normal and advanced breast and lung malignant tissue were also analysed using the gas chromatography-mass spectrometry (GC-MS) that is a highly accurate analytical method but it is too slow and costly to use in any routine diagnostic procedure.

Both breast and lung malignancy can be detected and distinguished at an early stage using three basic physical parameters of the pulse such as amplitude, rise time and fall time and also using derived parameters such as FWHM, area of the pulse, ionization cluster size, and ion drift time those are recorded by the detector in presence of VOCs exhaled by the samples over a range of pressure (1 to 10 Torr). In addition, frequency of the pulse plays a vital role as it is varied enormously among the stages of breast and lung malignancy and also between the two types of malignancy. However, it requires a constant pressure between 1 to 10 Torr. Based on these, it is confirmed that the present 3D positive ion detector can be used to detect both breast and lung malignancy and also to distinguish them. To strengthen the present results, the GCMS analysis confirmed that the VOCs exhaled by the breast and lung malignancy are different in terms of type of VOC, number of VOCs and its abundance.

The present investigation gives an opportunity to serve human society by making the present 3D positive ion detector as an economic, accurate, reproducible, user- friendly, portable, reliable, and non-invasive tool to detect breast and lung malignancy at an early stage by analysing the breath samples exhaled by normal and abnormal people.
